# Translation from the Spanish Made for the Examiner

**Published:** 1854-08

**Authors:** 


*Translation from the Spanish made for the Examiner.*

Verraricio Moreno y Lopez of Valtierra (Navarra) relates in the "El Porvenir Medico," of March 10, 1854, that among ignorant midwives it is common *to milk* forcibly both mother and child immediately after its birth. As soon as separated from the cord, the midwife takes the new born infant into her lap, and strongly compresses its mammæ betwixt her fingers until a few drops of serosity are forced from them, regardless of the cries of the new-born sufferer. The notion is, that this serosity is a deposit of milk which must be expelled in order to prevent the child from becoming sick, and to secure for the mother milk fever, which, without this operation, would fail.

"The skin, cellular tissue, muscles, glands, arteries, veins, lymphatic vessels, and even the nerves have I seen diseased in consequence of the forcible compressions made by the midwife; so that no one should be surprised if I suspect this practice as a cause of chronic swellings of the mammary gland in girls of 13 or 14 years old." "I am sometimes told, when consulted for considerable inflammation in the breasts of infants, that it followed in consequence of not having been well milked at the time of birth by the midwife."

In the same number, José Duch of Centillas (Barcelona) in order to show the importance of the obstetric art, relates six cases of difficult labor, analogous to each other. Three were left to natural efforts, and three were assisted; in the three first, vesico-vaginal fistulæ occurred, and in the others the patients happily recovered.

The first case was of a woman who was delivered of her first child in 1846 after a tedious and painful labor; the head remained impacted in the inferior strait during 24 hours, and the bladder was not relieved during the time, but the function was well performed soon after the birth, and through the day, but from that time urine flowed involuntarily and continuously. It was ascertained by examination with a sound that a hole, capable of receiving the pulp of the finger, existed between the bladder and vagina. Forceps had not been used. The patient refused all treatment, and to this day suffers from vesico-vaginal fistula.

The second case was of a French woman 20 years of age, to whom the relator was called after she had been more than a day in labor, (with the first child,) and after the head had descended into the inferior strait several hours. Examination proved the presentation to be the mento-sacral of Baudeloque. The head was unusually large, its ossification well advanced; teeth were perceived in the examination. The patient had not urinated since the preceding day; from the pressure of the head of the fœtus on the neck of the bladder, vesico-vaginal fistula was prognosticated. By introducing two fingers into the mouth, the chin was depressed, and by traction, aided by a forcible pain, delivery was accomplished, but not without tearing the perineum.

A vesico-vaginal fistula at the neck of the bladder formed. By constantly wearing a gum elastic sound, with a receptacle for the urine at its free extremity, (renewed from time to time as occasion required,) and remaining quiet, the patient was completely cured at the end of some months.

The third case happened in 1847. Maria Terré, aged 24, of robust and sanguine temperament, was taken in her third labor. The breast presented with the head towards the pubis, and both arms were extruded. The pains had been strong since the preceding day, but ineffectual.

The parturient had not urinated for very many hours, and was suffering a dull pain in the pubic region. Turning was proposed, but the woman most obstinately refused to submit to manipulation of any kind.

The pubic pain continued; urine was not expelled; and catheterism was impracticable. Death was prognosticated if the patient longer refused assistance, and at night fall, when turning could not be easily accomplished under the circumstances, she yielded. Embriotomy was practised.

After the extraction of the fœtus the patient was calm, urinated, and rested awhile. In spite of the most rigid treatment, pain in the lower belly, with fever, thirst, and tension over the region of the bladder, recurred on the following day.

The diagnosis was cystitis, consequent upon the pressure the organ had suffered. Leeches and other antiphlogistic means were used. After the pains ceased, the lochia, which had flowed regularly, suddenly increased, exhaling the peculiar fœtor of gangrene.

The symptoms changed; the face became pale, the pulse low and the general sinking and cold sweat indicated approaching dissolution; but from the natural strength of constitution, a favorable reaction ensued, and the patient complained of the genitalia and anus. A part of the posterior parietes of the bladder adhered to the anterior wall of the vagina. The next day a slough was discharged, converting the bladder and vagina into a single cavity.

This woman suffered much afterwards from excoriations caused by the constantly escaping urine. She has had no more children, but she still lives, enjoying robust health.

The fourth case was one of head presentation in a pelvis narrowed by exostosis. Craniotomy was practised, and the mother did well.

In the fifth case, in which the body presented with the left arm extruded, delivery was effected by turning.

The sixth case was a first labor in a woman of 20 years old. The head in the right occipito-cotoloid position had descended into the inferior strait. Uterine contractions had ceased, and ergot failed to reinduce them. The woman had not urinated for more than twelve hours, and the urethra was so firmly compressed between the child's head and the arch of the pubis that catheterism was impossible.

"It was a case for the application of forceps, but I did not possess the instruments. Poor village doctors, who exist amidst privations, are scarcely able to pay subscription for a scientific periodical to keep them instructed in the progress of knowledge, cannot afford to keep an arsenal of instruments, and at the same time maintain a family."

Under the circumstances, the doctor resorted to craniotomy and saved the mother. He expresses an opinion that had not delivery been artificially effected in the last three cases at the proper time, vesico-vaginal fistula would have resulted.

